# O4-4 Relation between physical activity level and environment perception among older adults

**DOI:** 10.1093/eurpub/ckac094.028

**Published:** 2022-08-29

**Authors:** Daniela Lopes dos Santos, Temistocles Vicente Pereira Barros, Nicanor Dornelles, Barbara Sutil da Silva, Patricia Fagundes Soares, Leonardo Backes, Mateus Giacomini

**Affiliations:** Physical Education and Sports Center, Federal University of Santa Maria, Santa Maria, Brazil

**Keywords:** physical activity, environment, perception, older adults, health

## Abstract

**Introduction:**

Regular physical activity (PA) is associated to a decrease in morbidity and mortality and to a better quality of life. The environment has been shown as an important factor in the adoption of an active lifestyle, especially among the elderly, since it may be the difference between a dependent or independent living and a better health status. Thus, the purpose of this study was to analyze the relation between the PA level and the environment perception of the elderly from a city in southern Brazil.

**Methods:**

Three neighborhoods of the city of Santa Maria, RS - Brazil were drawn to be part of the investigation, with low, medium and high socioeconomic levels. The sample had 202 subjects. An adapted version of the International Physical Activity Questionnaire for the Brazilian elderly (Mazo & Benedetti, 2010) was used to evaluate PA level and a Portuguese version of the Neighborhood Environmental Walkability Scale (Salvador et al, 2009) to evaluate environment perception. The statistical analyses were done by relative and absolute frequencies and logistic regression.

**Results:**

The prevalence of adequate PA levels was inferior to 30% in the three studied neighborhoods and the average of minutes spent with PA per week was 63.14 (±115.15). From the 41 variables considered in the environment perception, four had a p > 0.20 being selected for the multiple logistic regression model. The presences of fair and pedestrian crosswalks near home were the most influent factors on PA: the presence of fair increases 3.3 times the chance of the elderly being physically active and the crosswalks increase 2.7 times that chance.

**Conclusion:**

A low prevalence of physically active elderly was observed in Santa Maria, which was also shown in other studies with similar samples. The variables that most positively influenced the older adults' PA levels were the presence of fair and pedestrian crosswalks, showing the importance of the creation and maintenance of environments that facilitate PA, as well as public policies that promote healthy and safe environments.

